# Data Augmentation of Surface Electromyography for Hand Gesture Recognition

**DOI:** 10.3390/s20174892

**Published:** 2020-08-29

**Authors:** Panagiotis Tsinganos, Bruno Cornelis, Jan Cornelis, Bart Jansen, Athanassios Skodras

**Affiliations:** 1 Department of Electrical and Computer Engineering, University of Patras, 26504 Patras, Greece; skodras@upatras.gr; 2 Department of Electronics and Informatics, Vrije Universiteit Brussel, 1050 Brussels, Belgium; bcorneli@etrovub.be (B.C.); jpcornel@etrovub.be (J.C.); bjansen@etrovub.be (B.J.); 3 IMEC, 3001 Leuven, Belgium

**Keywords:** electromyography, data augmentation, deep learning, CNN, sEMG, hand gesture recognition

## Abstract

The range of applications of electromyography-based gesture recognition has increased over the last years. A common problem regularly encountered in literature is the inadequate data availability. Data augmentation, which aims at generating new synthetic data from the existing ones, is the most common approach to deal with this data shortage in other research domains. In the case of surface electromyography (sEMG) signals, there is limited research in augmentation methods and quite regularly the results differ between available studies. In this work, we provide a detailed evaluation of existing (i.e., additive noise, overlapping windows) and novel (i.e., magnitude warping, wavelet decomposition, synthetic sEMG models) strategies of data augmentation for electromyography signals. A set of metrics (i.e., classification accuracy, silhouette score, and Davies–Bouldin index) and visualizations help with the assessment and provides insights about their performance. Methods like signal magnitude warping and wavelet decomposition yield considerable increase (up to 16%) in classification accuracy across two benchmark datasets. Particularly, a significant improvement of 1% in the classification accuracy of the state-of-the-art model in hand gesture recognition is achieved.

## 1. Introduction

In the era of Deep Learning (DL), research in many domains has shown that bigger datasets can result in better models, since, in general, training on more data allows better generalization. The problem with some small datasets is that they have been collected under different conditions, thus merging them is not an option. When a task involves the analysis of biomedical signals, e.g., electroencephalography (EEG), electromyography (EMG), electrocardiography (ECG), etc., acquiring bigger datasets is a complicated task, and it can be an unpleasant experience for the patients due to tiredness and patient’s limitations or physical impairments. In addition, manual effort might be needed for cleaning and labeling the data. Consequently, the absence of a sufficient amount of data makes analyzing these signals quite a challenging task. In this work, we address the problem of limited data for the task of hand gesture recognition based on surface electromyography (sEMG) using data augmentation—a promising approach for enhancing existing datasets, which could allow further research and analysis.

Data augmentation comprises a set of approaches that aim at inflating the amount and diversity of available data for training models for a target task without the need to collect new data. This family of techniques builds synthetic/artificial data by transforming existing labeled samples so as to help the model learn the range of intra-class variation that one could possibly observe. A key challenge for data augmentation is to generate new data that maintain the correct label. In many tasks, this requires domain knowledge, which in the case of sEMG can be difficult to exploit due to high within-subject variability (i.e., the same person can perform the same gesture in many different ways, while factors such as fatigue and sweat affect the properties of the recorded signal). Data augmentation is also one of the approaches to deal with the problem of overfitting because the augmented data are expected to represent a more extensive set of possible data points, thus reducing the dissimilarity between the training and validation/testing sets.

Over the last few years, the subject of data augmentation has attracted many researchers and considerable progress has been made especially in the domain of computer vision, which influences many other research areas. Augmentation methods can be divided into two big categories [1]: Basic image manipulations (e.g., kernel filters, geometric and color transformations [2]) and DL approaches (e.g., neural style transfer [3] and Generative Adversarial Network (GAN) [4,5]). Furthermore, meta-learning methods (e.g., Smart Augmentation [6], AutoAugment [7]) can utilize neural networks in order to learn a set of appropriate augmentations for a given dataset and task, while the RandAugment strategy [8] manages to reduce the augmentation search space without reducing the performance of the model.

In the area of biosignal processing, augmentation methods have been developed as well. In the work of [9], a set of simple sequence manipulations (e.g., warping, time shuffling, additive noise) are evaluated on a Parkinson’s disease dataset recorded with accelerometer sensors. For brain computer interfaces, [10] investigates the possibility of electrode shifts of an EEG cap as a means of generating augmented data that correspond to spatial distortions. An extensive review of augmentation methods for EEG-related tasks is performed in [11]. The authors evaluate a wide range of augmentations that includes data manipulations such as additive noise and sliding windows as well as deep learning approaches based on GANs. Their results showed that additive noise and sliding windows provided the highest accuracy increase across different tasks. Furthermore, the authors of [12] have developed a method based on sub-optimally aligned sequences for generating augmented data. Their approach evaluated across different datasets, including EEG and ECG signals, yielded better or equivalent performance compared to existing methods.

Various augmentation strategies have been applied to sEMG-based gesture recognition. Basic data manipulations like the addition of Gaussian noise [13,14] and the electrode shift of a high density electrode grid [15] have provided limited gains. In [16], a set of domain specific augmentations are evaluated. These include the simulation of sensor noise, electrode displacement, and fatigue. Additionally, a sliding window approach is used instead of non-overlapping signal segments. Their analysis showed that the sliding window approach results in significant improvement, while the additive noise performed the worst. The fact that domain-specific approaches like electrode displacement and muscle fatigue approximation failed to improve the classification accuracy confirms the difficulty of generating appropriate sEMG signals. On the other hand, electrode displacement has been successful as an augmentation method for the problem of user authentication based on sEMG. The authors of [17] apply a circular rotation of the eight electrodes of the Myo armband, thus expanding the original data up to 8×. In this case, the electrode displacement augmentation improves the performance of the model because the enhanced dataset still belongs to one person, while a specific hand gesture is not required for the successful authentication. Finally, DL-based approaches have shown great potential for enhancing EMG datasets. In [18], the authors utilize a GAN architecture combined with Style Transfer to augment sEMG signals recorded from Parkinson’s disease patients. Specifically, their model learns the tremor patterns of a patient and applies them to signals acquired from healthy subjects. Thus, they can use able-bodied subjects to investigate how patients’ movements are affected by the disease.

Despite the research progress in many fields, there are very few studies that provide an extensive evaluation of the augmentation methods applied to a specific task. In this work, we investigate the application of data augmentation to the problem of hand gesture recognition based on sEMG signals, while providing a thorough assessment of the proposed methods. The training dataset is enhanced with synthetic data in order to improve the recognition accuracy of Convolutional Neural Network (CNN) classifiers. The main contributions are:

- the presentation of common (i.e., additive noise, overlapping windows) and novel (i.e., magnitude warping, wavelet decomposition, synthetic sEMG models) data augmentation methods for sEMG signals
- an extensive and systematic evaluation of the aforementioned augmentation methods
- an improvement in classification accuracy of the state-of-the-art model [19].

The rest of the paper is organized as follows. In Section 2, we give the details of the proposed approaches and the CNN architectures used in the experimentation. The experiments are presented in Section 3. Next, the results are shown in Section 4 and the discussion follows in Section 5. Finally, Section 6 summarizes the outcomes and outlines future work.

## 2. Materials and Methods

### 2.1. Augmentation Tools

The purpose of data augmentation is to generate a new labeled signal $\left\{x_{i}^{*},y_{i}^{*}\right\}$ from a real one $\left\{x_{i},y_{i}\right\}$ without altering the class label, i.e., $y_{i}=y_{i}^{*}$. In this work, five data augmentation methods are applied to the RMS signals of sEMG sequences. The motivation for choosing these five methods was to include the most common ones encountered in the literature (i.e., additive noise), as well as a variety of different methods that take into account both time-domain and frequency-domain characteristics of the signal. Their comparison is made with the CNN architectures described in Section 2.2 across the datasets presented in Section 2.3.

As in previous works [13,16], emulating additive sensor noise can be achieved by the addition of *Gaussian Noise (GN)* with a desired signal-to-noise ratio (SNR). A noise signal *n* is sampled from a Gaussian distribution with zero mean and variance equal to the ratio of the signal power over the SNR:

(1) $$\begin{array}{cc} x_{i}^{*} & =x_{i}+n\\ n & ∼N(\mu =0,\sigma =\sqrt{\frac{x_{i}^{2}}{\mathrm{SNR}}}) \end{array}$$

**Figure A1:**
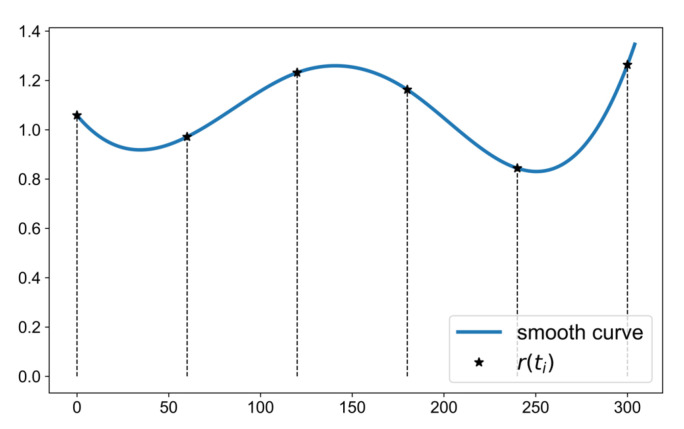
Example of smooth curve generation used in the MW method. Random values for T=6 equidistant points are sampled from N(μ=1,σ=0.2) and then interpolated using a cubic spline.

*Magnitude warping (MW)*, used in [9] to change the shape of accelerometer signals for the motion recognition task, is applied in a similar fashion in this work to deform the sEMG amplitude. This is achieved through the multiplication of the signal with a random smooth curve of small variance, selected empirically, and a mean equal to one. This smooth curve is generated by fitting a cubic spline curve on a few random samples. Firstly, *T* equidistant time points, $t=\{t_{1},\;\ldots \;,t_{T}\}$, are selected. Then, for each one a random number, $r(t_{i})$, is sampled from a normal distribution with unity mean and an interpolation with cubic splines is applied, as shown in Figure A1. Finally, the augmented signal is obtained by the elementwise product of this interpolated curve (CubicSpline(r)) with the sEMG signal $x_{i}$:

(2) $$\begin{array}{cc} x_{i}^{*} & =x_{i}\cdot \mathrm{CubicSpline}\left(r\right)\\ r & =\{r\left(t_{1}\right),\;\ldots \;,r\left(t_{T}\right)\}\\ r(t_{i}) & ∼N(\mu =1,\sigma ) \end{array}$$

Filtering with different types of filters can generate various views of the input signal. Here, we apply *Wavelet Decomposition (WD)* as an augmentation method using a randomly selected wavelet ψ and decomposition level *l*, where all detail coefficients (cD) extracted through the Discrete Wavelet Transform (DWT) are modified by a factor *b*, and the approximation coefficients (cA) are not altered. Then, the Inverse DWT (IDWT) is used to obtain the augmented signal:

(3) $$\begin{array}{cc} x_{i}^{*} & =\mathrm{IDWT}(\mathrm{cA},{\mathrm{cD}}^{*},\psi )\\ {\mathrm{cD}}^{*} & =b\cdot \mathrm{cD}\\ \mathrm{cA},\mathrm{cD} & =\mathrm{DWT}(x_{i},\psi ,l) \end{array}$$

Furthermore, an sEMG simulation model [20] is applied to generate synthetic data. For a single electrode, the method applies a shaping filter *g* to a random vector *w* followed by multiplication with an amplitude signal α and the addition of Gaussian noise *n*. The frequency characteristics of the generated signal are determined by the filter *g*, the Power Spectral Density (PSD) of which depends on the two frequency values, $f_{l}$ and $f_{h}$. To account for the correlation between adjacent electrodes, we extend the model of [20] by sampling *w* from a multivariate Gaussian of zero mean, and a covariance matrix computed from the correlation between the electrode signals. As α, we use the lowpass filtered (LPF) signal of the real sEMG calculated by a moving average filter over 15 ms windows. We call this method *sEMG Simulation (SS1)*:

(4) $$\begin{array}{cc} x_{i}^{*} & =(w*g)\alpha +n\\ w & ∼N(\mu =0,\sigma =\mathrm{Cov}\left(x_{i}\right))\\ {\mathrm{PSD}}_{g}\left(f\right) & =\frac{f_{h}^{2}f^{2}}{\left(f^{2}+f_{l}^{2}\right){\left(f^{2}+f_{h}^{2}\right)}^{2}}\\ n & ∼N(\mu =0,\sigma =0.1)\\ \alpha & =\mathrm{LPF}(x_{i}) \end{array}$$

A similar simulation model has been developed by [21]. In their approach, the sEMG variance (${\bar{\sigma }}^{2}$) is generated from a probability distribution with a shape determined by the muscle activations. The inverse gamma distribution, IG(α,β), is used with parameters estimated by the expectation maximization algorithm (EM) from the real sEMG signals. Shaping filter *g* and noise *n* are calculated as in Equation (4). We denote this method as *sEMG Simulation (SS2)*:

(5) $$\begin{array}{cc} x_{i}^{*} & =\left(w*g\right)\sqrt{{\bar{\sigma }}^{2}+n}\\ w & ∼N(\mu =0,\sigma =1)\\ {\bar{\sigma }}^{2} & ∼\mathrm{IG}(\alpha ,\beta )\\ \alpha ,\beta & =\mathrm{EM}(x_{i}) \end{array}$$

Finally, a random combination of the above methods is explored, indicated as *Augmentor*. Given there are *K* augmentations available, we can distinguish between three approaches:

1. *Augmentor.One (AO)*: applies only one augmentation method randomly selected from the available methods, e.g., if we have two augmentations ($A_{1},A_{2}$), this approach is equal to applying $A_{1}$ to half of the data and $A_{2}$ to the other half.
2. *Augmentor.All (AA)*: applies all the *K* methods consecutively, i.e., $x_{i}^{*}=A_{K}\left(A_{K-1}\left(\ldots A_{1}\left(x_{i}\right)\right)\right)$.
3. *Augmentor.Random (AR)*: applies each of the *K* augmentation methods successively with a probability $p_{k}$, k∈[1,K]: $x_{i}^{*}=A_{K}\left(p_{K},A_{K-1}\left(p_{K-1},\ldots A_{1}\left(p_{1},x_{i}\right)\right)\right)$. An augmentation method $A_{k}$ is applied if a random number sampled from a uniform distribution U(0,1) is greater than the threshold $p_{k}$. In these experiments, all threshold probabilities are equal to $p_{k}=0.5$.

**Figure A2:**
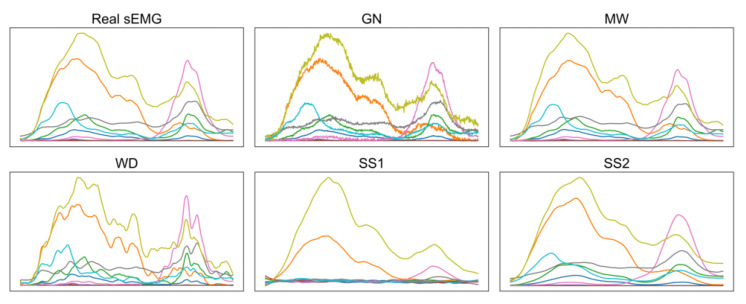
Examples of the investigated augmentation methods applied to the RMS of a sEMG signal from Ninapro-DB1. The colors correspond to the different electrode channels.

The order of the application in AA, AR approaches is: SSx-WD-MW-GN, where SSx is either SS1 or SS2. Examples of the generated signals for each method can be seen in Figure A2.

The final step of the augmentation pipeline consists of generating the input images using the *Sliding Window (SW)* method. This generates images of dimensions L×C, where *L* is the segment duration and *C* the number of sEMG electrodes, using a window step τ∈[1,L]. Contrary to the previous augmentations, SW does not generate new signals but instead it forms the input images with overlap (τ<L) or without (τ=L). Although in the majority of literature SW is not considered an augmentation method (except in [11,16]), we believe it is better to include it here, since it has many similarities with the crop augmentation used in computer vision [1].

Another variable of the proposed augmentation scheme is the amount of augmentation. This is controlled by the augmentation ratio, *R*, defined as the ratio of the number of generated signals over the number of the initial signals. For example, in the case of Ninapro-DB1, the training set consists of 52 gestures repeated seven times, i.e., 52×7=364 signals. Thus, an augmentation factor equal to nine means that in the augmented dataset there are 364×9=3276 generated signals and the training size (before segmentation) becomes 364+3276=3640. The appropriate value should be chosen in relation to the initial data size and the trainable parameters of the network.

### 2.2. CNN Architectures

The above-mentioned methods are evaluated by measuring the improvement of classification accuracy for two CNN architectures. These are a modified version of the simple model of [13] denoted as AtzoriNet* described in [22], and an implementation of the bigger network of [19] called *WeiNet* to evaluate how well augmentation works in overparameterized networks. Details of the models can be seen in Table A1.

The version of *AtzoriNet* presented in [13] consists of blocks of convolutional and average pooling layers that end in a single softmax layer. The outcome of our experimentation in [22] is a model with improved accuracy that includes max pooling and dropout layers.

*WeiNet* is described as a multi-stream approach where a different convolutional pipeline is applied to streams of the input data. Each data stream is processed by two convolutional and two locally connected layers that do not decrease the spatial resolution of the input. The outputs from each stream are merged into a single feature vector via concatenation. Then, this feature vector passes through a series of fully connected layers followed by a softmax classifier.

### 2.3. Datasets

Augmentation methods are evaluated across two datasets: Ninapro-DB1 [23,24] and putEMG [25]. Ninapro-DB1 consists of 27 healthy subjects performing 53 gestures, each one repeated 10 times, recorded with 10 electrodes. The putEMG dataset consists of data from 44 subjects performing eight gestures, where each gesture is repeated 20 times during three tasks (denoted as ‘repeats-long’, ‘sequential’, and ‘repeats-short’), recorded with 24 electrodes arranged in three rows. In the following experiments, only the first task (eight repetitions) of putEMG is used. We have not used the recordings of ‘sequential’ and ‘repeats-short’ tasks because we have opted to follow the classical approach of repeating the same gesture as in Ninapro-DB1 and in most of the studies on hand gesture recognition. Details of the datasets can be found in Table A3. To avoid segments of the same signal being in both training and validation sets, the partition of data into training, validation and testing sets is based on the initial signal repetitions, e.g., segments generated from repetition 5 of the Ninapro-DB1 can be found in the test set only. A complete description is given in Table A3. In addition, a variance was observed at the training set regarding the amount of segments per class due to the inconsistency in gesture duration. Hence, this class imbalance was solved by randomly removing segments from the over-represented gestures.

For the preprocessing of the signals, we follow the guidelines for the corresponding dataset [13,25]. For Ninapro, a 1st order Butterworth lowpass filter with cut-off frequency at 1 Hz is applied to the RMS. For putEMG, firstly a notch filter at 50 Hz followed by a 5th order bandpass Butterworth filter [20 Hz, 700 Hz] are applied. Then, to be consistent with the preprocessing in Ninapro, the RMS is calculated over 100 ms windows and the same lowpass filter is applied. Finally, to eliminate any effects of the sampling frequency to the model architecture, the sEMG signals are subsampled to the lowest sampling frequency between the datasets (i.e., 100 Hz).

## 3. Experiments

The first step of the experimentation consists of determining the proper values for the parameters of the augmentation methods. This is achieved through a grid search performed for the AtzoriNet* on each dataset separately. Specifically for the WD, MW, and GN methods, the values shown in Table A4 are evaluated. In the case of WD, the ψ and *l* correspond to the mother wavelet and the decomposition level used to compute the wavelet coefficients cA and cD (i.e., average and details coefficients, respectively), while *b* is the multiplication factor that modifies the details coefficients (Equation (3)). In the cases of MW and GN, there is a single hyper-parameter that controls the variance, σ, of the smoothing curve (Equation (2)) and the amount of additive noise, characterised by SNR (Equation (1)), respectively. For these experiments, only the first 20 subjects are used. During training the validation repetitions shown in Table A3 are held out from the training set.

At a next step, two experiments are carried out: (a) the evaluation of the augmentation methods with the optimized augmentations using the complete datasets, and (b) the improvement of the state-of-the-art model (*WeiNet*). In the first case (a), the augmentation methods (i.e., SS1, SS2, WD, MW, GN) are compared against each other, and in the latter (b) the methods that performed best are applied to the *WeiNet* model. For the Augmentor methods, the top-3 best performing augmentations as evaluated on Ninapro-DB1 are used setting $p_{k}=0.5$ in the case of AR.

For the AtzoriNet* model, the input images of size L×C, where *L* the segment duration and *C* the number of electrodes, are generated using 150 ms segments, i.e., L=15. In the augmentation hyper-parameter selection step, the images are generated without overlap, i.e., τ=L, and the size of the augmented training set is 10 times the size of the original, i.e., R=9. For the rest of the experiments, window overlaps τ={15,8,4,2,1} and augmentation ratios R={1,4,9,15} are evaluated.

In the case of *WeiNet*, the input images are generated using 200 ms segments and maximum overlap, i.e., L=20,τ=1, as proposed by the authors of the model. Window overlap is held constant for the investigated augmentation ratios R={1,4,9}.

Augmentation methods are indicated by their name with the window step in parentheses. For example, SS1(15) corresponds to SS1 augmentation with window step τ=15 (no overlap), while MW(01) is the MW method with maximum overlap. In the following experimentation, we consider as a first baseline the case without any augmentation (R=0) and window step τ=L denoted as SW(15)(baseline-1). Taking into account that there is no consensus in literature concerning the use of sliding windows (SW) as an augmentation method, we also consider the maximum overlap approach SW(01) as a second baseline, i.e., SW(01)(baseline-2).

Data preparation and training of the models are carried out on a workstation with an Intel i9-7920X 2.90 GHz processor, 128 GB RAM and an Nvidia GeForce RTX 2080Ti 11GB GPU. The implementation is based on the Tensorflow Python library.

### 3.1. Metrics

The performance metrics used for the evaluation are the accuracy and the loss values computed on the validation/test set averaged across the subjects. Specifically, the metrics values for a given dataset, CNN model and augmentation method are compared with the corresponding values of the other methods as well as the baseline of the same dataset and model. Additionally, the average across subjects f1-score for each gesture is shown. Statistical significance is analysed through an Analysis of Variance (ANOVA) followed by post-hoc pairwise tests. To assess the quality of the generated data, a low dimensional tSNE embedding [26] of the CNN features computed on the real and the augmented sEMGs is provided. Furthermore, metrics that evaluate the gesture clusters in the high-dimensional feature space are provided. These are:

- the Silhouette Coefficient (SC) [27]:
  $$\mathrm{SC}=\frac{1}{N}\sum_{i=1}^{N}\frac{b(i)-a(i)}{max\{a(i),b(i)\}}$$
  where *N* is the total number of points, a(i) is the mean distance between point *i* and all other data points in the same cluster and b(i) is the smallest mean distance of *i* to all points in any other cluster, of which *i* is not a member.
- the Davies–Bouldin (DB) index [28]:
  $$\mathrm{DB}=\frac{1}{K}\sum_{i=1}^{K}max_{j\neq i}\left(\frac{\sigma_{i}+\sigma_{j}}{d(c_{i},c_{j})}\right)$$
  where *K* is the number of clusters, $\sigma_{i}$ the average distance of all points in cluster *i* from the cluster centroid $c_{i}$ and $d(c_{i},c_{j})$ the distance between the two centroids.

SC values are in the range [−1, 1], where a high value indicates that the points are well matched to their own clusters. DB index values are positive numbers and the optimal clustering solution has the smallest DB value.

### 3.2. Model Optimization

Optimization hyper-parameters for the two CNN models are reported in Table A2. Specifically, the AtzoriNet* models were trained with the Adam [29] optimizer using a constant learning rate of 0.001 and a batch size of 512 for 100 epochs. Furthermore, to avoid overfitting the models, an early stopping strategy was adopted and a weight decay ($l_{2}$) regularization of 0.0005 was used. In the case of *WeiNet*, the networks were optimized following the guidelines of the authors [19], i.e., using stochastic gradient descent (SGD) with momentum for 28 epochs with a learning rate of 0.1 divided by 10 after epochs 16 and 24, a batch size of 1024 and $l_{2}$ regularization equal to 0.0001.

## 4. Results

The augmentation hyper-parameters search space along with the selected values are shown in Table A4 and the average accuracy on the validation set is shown in Table A5. The parameter values that scored the highest accuracy in Ninapro-DB1 were as follows: for GN, the SNR=30, for MW, the σ=0.1 and for WD, the ψ=sym4, l=5, b=3. In putEMG, the corresponding values were: SNR=35, σ=0.1 and ψ=db7, l=5, b=0.

**Figure A3:**
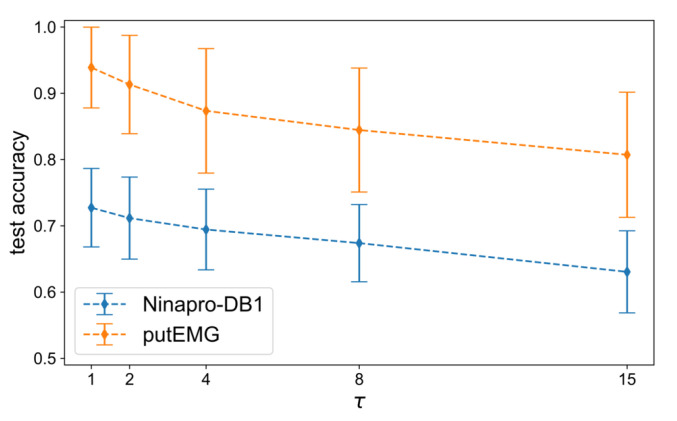
Average test accuracy of the AtzoriNet* for different sliding window steps τ.

**Figure A4:**
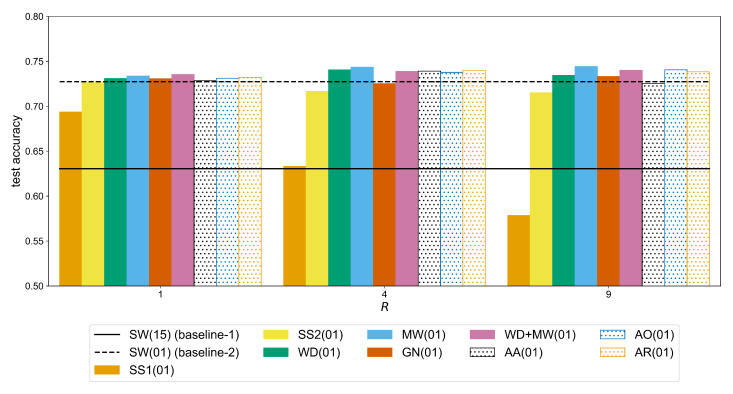
Average test accuracy of AtzoriNet* for each method at different augmentation ratios, *R*, for the Ninapro-DB1 dataset. The solid line corresponds to τ=15 and the dashed line to τ=1 (see also Figure A3).

**Figure A5:**
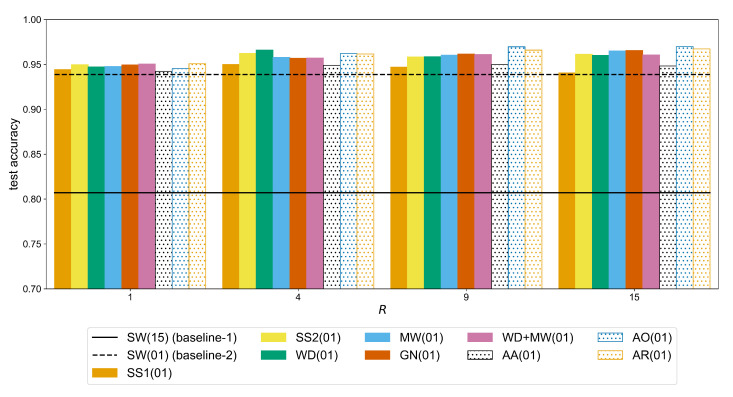
Average test accuracy of AtzoriNet* for each method at different augmentation ratios, *R*, for the putEMG dataset. The solid line corresponds to τ=15 and the dashed line to τ=1 (see also Figure A3).

**Figure A6:**
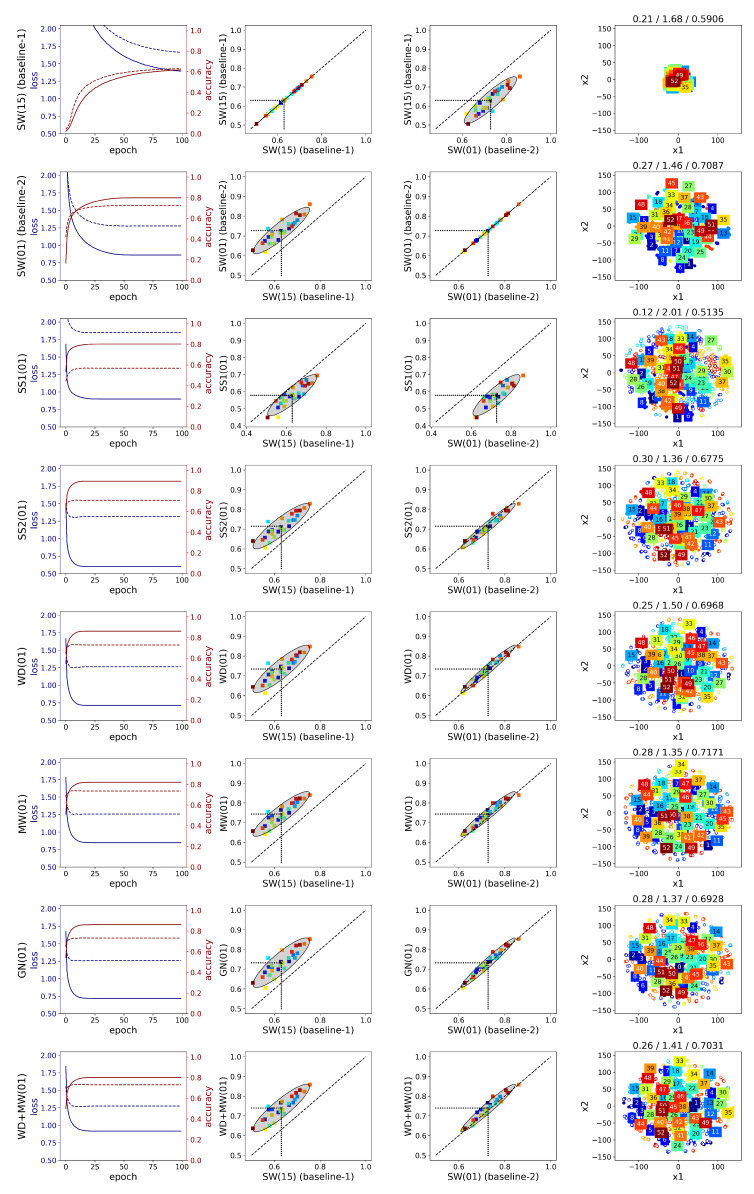
Average loss and accuracy graphs (**left**), augmentation vs. baseline-1 and baseline-2 accuracy per subject (**middle**), and t-SNE embeddings of a single subject (**right**) for the different augmentation methods on Ninapro-DB1. The investigated method is shown on the left of the figure. In the left plot, the solid lines correspond to training, while the dashed lines to testing. In the middle plots, color points correspond to different subjects and the ellipse shows the general shape of the points. In the t-SNE visualizations, color points correspond to different gestures, while the values at the title are the SC/DB/accuracy metrics.

**Figure A7:**
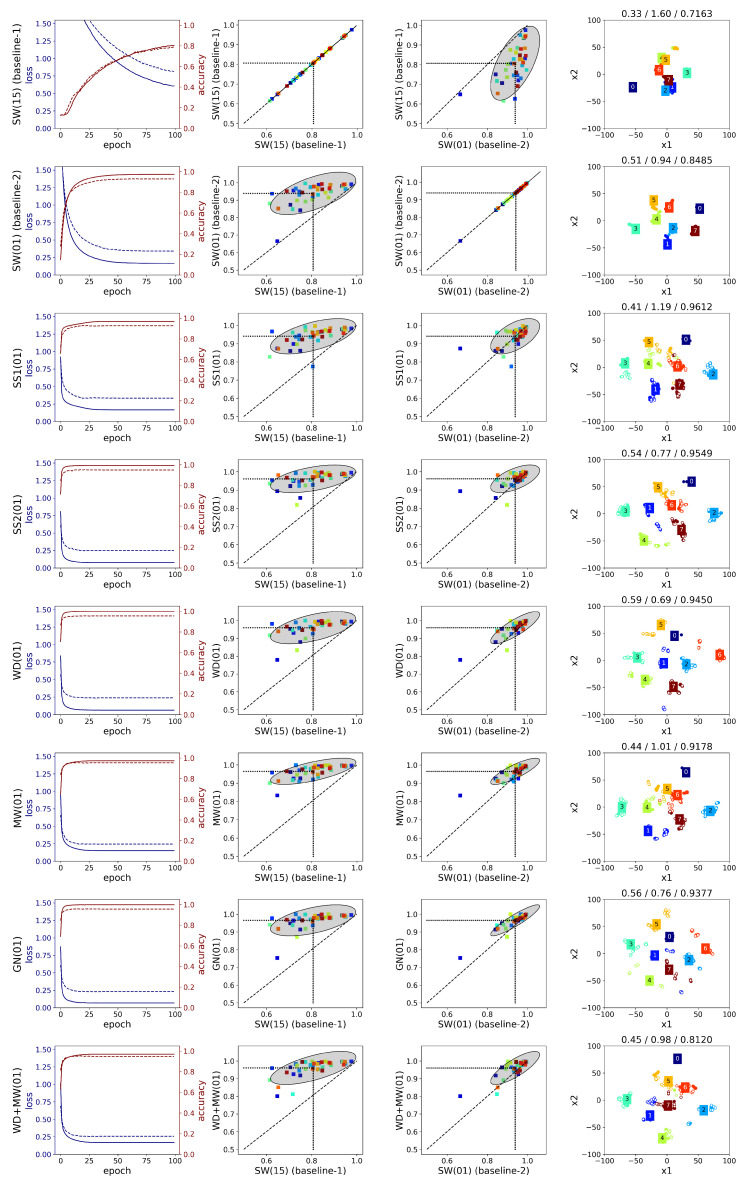
Average loss and accuracy graphs **(left)**, augmentation vs. baseline-1 and baseline-2 accuracy per subject (**middle**), and t-SNE embeddings of a single subject (**right**) for the different augmentation methods on putEMG. The investigated method is shown on the left of the figure. In the left plot, the solid lines correspond to training, while the dashed lines to testing. In the middle plots, color points correspond to different subjects and the ellipse shows the general shape of the points. In the t-SNE visualizations, color points correspond to different gestures, while the values at the title are the SC/DB/accuracy metrics.

**Figure A8:**
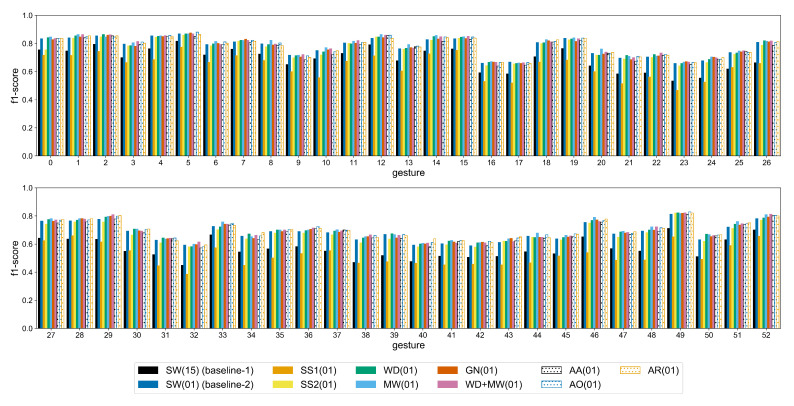
f1 scores of the AtzoriNet* for each gesture of Ninapro-DB1 dataset for τ=1 and R=9.

**Figure A9:**
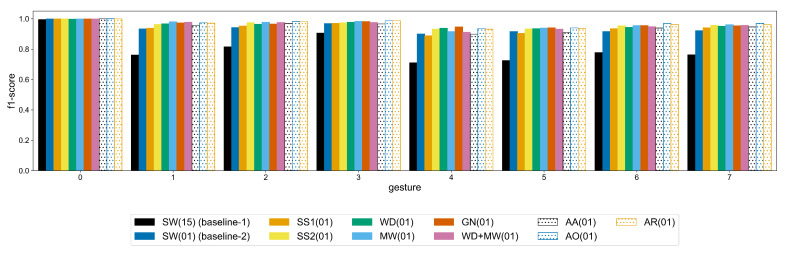
f1 scores of the AtzoriNet* for each gesture of putEMG dataset for τ=1 and R=15.

The evaluation of the augmentation methods on the AtzoriNet* [22] across the two datasets is as follows: Figure A3 shows the average accuracy when different overlap steps are used for the generation of the images without any other augmentation applied. Figure A4 and Figure A5 and Table A6 and Table A7 contain the accuracy metrics for each augmentation method evaluated on the test set of the two datasets. Figure A6 and Figure A7 show the accuracy and loss graphs during training and testing (left), the performance for each subject with and without augmentation (middle), and the 2D embedding of the CNN features along with the corresponding clustering metrics computed on the augmented training set and the test accuracy of the most representative subject (right). The loss graphs show the loss and accuracy values during training and testing averaged across subjects. The improvement for each subject is visualized as a scatter plot of the accuracy of an augmentation method vs. the baseline-1 and baseline-2 approaches. The shape and orientation of the ellipses show how the augmentation methods affect the model accuracy compared to the baseline. When the classification variance (i.e., the spread of the data points) is equal between the corresponding baseline and the augmentation method, the point cloud is parallel to the main diagonal. In addition, a point cloud above the diagonal means that the classification accuracy is improved by applying the corresponding augmentation. CNN features extracted from the last convolutional layer of the model of a single subject are visualized using t-SNE—an algorithm for dimensionality reduction that enables the embedding of high-dimensional data into a two-dimensional space for visualization purposes [26]. Empty circles correspond to features of the generated signals through augmentation, while gestures are represented with different colors. As a representative subject, we considered the one for which the clustering metrics were close to the average ones, found in Table A8 and Table A9 for Ninapro-DB1 and putEMG, respectively. The SC and DB metrics presented in Section 3.1 are calculated in the high-dimensional feature space and shown on the title of each t-SNE visualization along with the classification accuracy metric for that subject (i.e., SC/DB/accuracy). The average f1-score of each gesture on the test set is shown in Figure A8 and Figure A9. Finally, Table A10 and Table A11 show the results of the paired *t*-tests on the classification accuracy for the smallest augmentation ratio (i.e., R = 1) and the largest one (i.e., R = 9 for Ninapro-DB1 and R = 15 for putEMG), while Table A12 and Table A13 the significance tests for the SC and DB clustering metrics on the two datasets.

Regarding the evaluation of the *WeiNet* [19], Table A14 shows the average accuracy of the augmentation methods on the Ninapro-DB1 dataset for τ=1, R=9. The analysis showed a significant improvement (p<0.001) when the MW augmentation is used.

## 5. Discussion

For the Discussion and the Conclusions sections, we always base the comparative performance analysis of the various methods on significance tests. No significant difference implies equal performance. In this discussion, we add to this quantitative approach some qualitative analyses of the behaviour of several methods on both datasets, supported by more intuitive reasoning or corroborated by different viewpoints.

The hyper-parameter selection conducted for the WD, MW, and GN augmentation methods provided some useful results (Table A5). For the SNR parameter of GN, a value of SNR=30 was selected by the greedy search, which is different compared to previous works on the *AtzoriNet* and Ninapro dataset [13,22]. In the case of the putEMG dataset, a higher SNR is chosen (SNR=35) for the GN augmentation since the signals in this dataset are already much noisier. The value of σ determines the degree of deformation applied to the sEMG, so a small value (σ=0.1) is selected to avoid synthetic signals that could represent a signal of a different gesture. In the case of WD, the parameter values are different in the two datasets.

The main evaluation of the methods is performed next, using the best hyper-parameters selected in the previous step. Firstly, we assess the effect of overlapping windows, since many works in the literature do not clarify whether it is used or not. The analysis of [16] showed that among the investigated methods the SW augmentation approach performed significantly better. In Figure A3, we can see the declining trend of the average accuracy when the window step, τ, increases. Specifically, with densely overlapping windows, the size of the training set increases more than with the augmentation ratio *R*, which translates to higher gain in classification accuracy. For example, when τ=15,R=0, there are 6038 training samples and when τ=15,R=9 there are 10× more samples, i.e., 60,380. On the other hand, when τ=1,R=0 the training size consists of 87,936 samples and when τ=1,R=9 there are 879,360. This confirms that using overlapping windows is an important factor of a DL pipeline for sEMG signal processing.

The second factor that affects the performance of the CNN model with respect to both baselines is the amount of augmentation expressed with the augmentation ratio *R*. In Figure A4, we can see that for the Ninapro-DB1 dataset a statistically significant improvement (observed through the following *p*-values, p=0.002 for GN, p=0.004 for WD+MW, p<0.001 for the remaining methods) can be measured for a ratio up to R=4. For example, the accuracy of the MW method (Table A6) improves from 0.7337 (R=1) to 0.7436 (R=4) (significant difference with a *p*-value of p<0.001), while the additional improvement when using R=9 is not significant (i.e., +0.0007 with *p*-value equal to p=0.728). In the case of putEMG (Figure A5), the accuracy continues to improve for bigger values of R. For the same augmentation method (MW), there is an increase from 0.9477 (R=1) to 0.9580 (R=4) (significant difference with a *p*-value of p=0.015) and then 0.9650 (R=15) (significant difference with a *p*-value of p=0.010). This is due to the fact that in putEMG there are less repetitions per gesture and also their duration is shorter than in Ninapro, so the number of sEMG windows is generally smaller. Therefore, a larger amount of augmented signals is required to train a given CNN model.

A detailed comparison is given below (Figure A6 and Figure A7) considering the following points: the average loss/accuracy graphs, the accuracy improvement per subject relative to the baselines (i.e., SW(15) and SW(01)), and the 2D embeddings. Regarding the use of overlapping windows, the loss graphs show that with maximum overlap the network weights are trained more efficiently. The performance for every subject is improved, though the high variance in the results remains as we can see from the spread of the points in the second column of Figure A6 and Figure A7. Additionally, the average SC and DB metrics for the SW(15) and SW(01) methods remain low. This is also illustrated in the embedding graphs which do not show any improvement in distinguishing the features of different gestures.

Comparing the two augmentation methods based on sEMG simulation, i.e., SS1 and SS2, we can see that the latter outperforms the former in both datasets regardless the value of augmentation ratio R. On the other hand, SS1 does not improve the accuracy in any of the subjects of Ninapro, since the ellipse in the second and third columns of Figure A6 is below the diagonal. An explanation for the difference in the performance of these two methods can be given by the corresponding clustering metrics of the CNN features (Table A8), where SS1 has the lowest SC value, i.e., 0.1535, and SS2 the highest, i.e., 0.2980 (the difference is significant with p<0.001 as shown in Table A12). These observations are visualized in the t-SNE graphs shown in the last column of Figure A6 and Figure A7 for a single subject. In the case of SS2, a few clusters of similar gestures appear, whereas the features of the augmented signals lie far away from the real data in SS1. As a result, when generating more augmented signals with SS1 by increasing the ratio R, the classification accuracy deteriorates rapidly (Table A6). In Ninapro, the accuracy deteriorates for bigger augmentation ratios when SS2 is used (the difference between R=1 and R=4 is significant with p<0.001), but for putEMG there is an improvement of 0.0124 from R=1 to R=4 (p=0.005). The difference between the two simulation methods lies in the calculation of the sEMG variance. In SS2, it is better estimated through an inverse Gaussian distribution using the EM algorithm. However, this leads to an increased computational demand which only affects the training time of the CNN.

In general, WD and MW followed by GN yield higher accuracy results so, these augmentations are considered in the Augmentor variants, i.e., AO, AA, AR. In Ninapro-DB1, the Augmentor methods are slightly below the other approaches while the best result is achieved with MW (Table A6). The WD and MW score higher accuracies in putEMG as well, but equally good results can be obtained with the AO and AR methods. Overall, the best performance in Ninapro-DB1 is achieved by the MW with an accuracy of 0.7443 (equally good to GN, WD+MW, AO, and AR (Table A10)), while in putEMG by the Augmentor AO with 0.9697 accuracy (equally good to other augmentations except SS1 where p<0.001 (Table A11)). When compared to SW(15) (baseline-1), these accuracy scores correspond to an improvement of over 11% on Ninapro (Table A6) and over 16% on putEMG (Table A7).

The learning curves averaged across subjects (first column of Figure A6 and Figure A7) can be used to assess the degree of overfitting by the difference between the training and testing curves. In the case of the Ninapro dataset (first column of Figure A6), MW performs better in reducing overfitting. On the other hand, in WD and GN, there is a great degree of overfitting, which can be explained by the fact that the features of the augmented signals remain close to the corresponding original ones. Although their combination, WD+MW, does not yield higher accuracy than MW alone, overfitting is further decreased. In general, from the first column of Figure A6, we may conclude that the investigated augmentations do not provide an adequate variety in the generated signals of *all* the 52 gestures in Ninapro-DB1, since the difference in the loss values between train and test is considerable. Similarly in the putEMG dataset, MW and WD+MW yield the best performance in terms of reduced overfitting (first column of Figure A7).

A few differences are observed between the learning curves of the two datasets. Specifically, in Ninapro, the gap between the training and testing loss curve is bigger than in putEMG. In addition, the final loss values are much lower in putEMG across every method. A possible explanation could be the fact that the classification task is easier in putEMG which contains less gestures compared to Ninapro-DB1. This is also depicted in the t-SNE embeddings (last column of Figure A6 and Figure A7) where some clear clusters are formed in the case of putEMG. Regardless of the differences in the learning curves, the behaviour of the augmentation methods is largely the same in the two datasets as described above.

A difference in the classification performance of the two datasets is observed in the middle plots of Figure A6 and Figure A7. For Ninapro (Figure A6), we observe a more consistent behaviour across the subjects since the accuracy variance in the augmentation methods (*y*-axis) is similar to the variance in the two baselines (*x*-axis), though a slightly lower variance is observed in the MW method. In the third column, we can see that almost all the subjects are above the diagonal when MW method is used, whereas in the case of SS2 the points are slightly below the diagonal. Furthermore, the classification accuracy changes the same way across the subjects. On the other hand, in putEMG (Figure A7), there is a high variance in the SW(15) (baseline-1) approach, which is minimized in the MW method. In addition, many subjects perform very well without any augmentation, thus there is a smaller margin for improvement in these cases compared to the subjects that score poorly without augmentation. This is more clear when the augmentation methods are compared to SW(01) (baseline-2), where the ellipses are less elongated and the angle between the ellipses and the diagonal is smaller. Overall, apart from the different hardware used in the two datasets, which has a dominant effect on the quality of the recorded signal, another reason that can explain these differences is the amount of gestures in the two datasets. Ninapro contains a huge variety of gestures where the subtle differences between them make the classification a much harder problem than in the case of putEMG with fewer gestures. The results of the augmentation methods on putEMG show that augmentation techniques are beneficial to improving the accuracy when there are too few data.

Regarding the clustering metrics and the t-SNE embeddings, the following can be observed. Between the two datasets, we see larger SC values and smaller DB values in putEMG (Table A9) compared to Ninapro-DB1 (Table A8). This is expected since the classification task is easier in putEMG, thus the CNN features of the same gesture can be clustered together as can be seen in the example t-SNE embeddings (last column of Figure A7). From the low dimensional visualizations of the CNN features for a single subject, we can see that for the augmentation methods with higher accuracy (e.g., SS2, GN), there is better separability between the clusters. This is in agreement with the cluster metrics, since the SC value is greater than in the baseline-1 and the DB is smaller (the differences are significant with p<0.001 in both datasets as shown in Table A12 and Table A14). Eventually, the average clustering metrics (Table A8) show significantly better separation (SC=0.2980,DB=1.36) of the SS2 method compared to all the other augmentation methods in the Ninapro dataset (the differences are significant with p<0.001 for the SC and p<0.05 for the DB metric (Table A12)). In putEMG, (Table A9), the AR augmentation offers better separation (SC=0.5394,DB=0.77) than the baselines, SS1, MW, WD+MW, and AA, but it is equally good to SS2, WD, GN, and AO (with *p*-values shown in the last column and last row of Table A13 for the SC and DB metric, respectively). Another observation is that in general there is an agreement between the SC/DB metrics and the accuracy score. For example, a high SC value corresponds to higher accuracy (Pearson’s correlation ρ=0.9418 with p<0.001 for Ninapro and ρ=0.8917 with p<0.001 for putEMG) and a large DB value to lower accuracy (Pearson’s correlation ρ=−0.9559 with p<0.001 for Ninapro and ρ=−0.9549 with p<0.001 for putEMG). However, ordering the augmentation methods with respect to metrics SC/DB might be different than a ranking based on the accuracy values (e.g., in Table A9, the AR method has the best values for the SC and DB metrics, but the highest accuracy is achieved by AO).

To assess the improvement for each individual gesture, the average f1-score is provided in Figure A8 and Figure A9. With the exception of the SS1 method in Ninapro, the remaining augmentation approaches improve the classification of every gesture. This improvement is mostly clear in the more complex gestures of Ninapro, e.g., labels 21–29 (wrist rotations) and labels 38, 40, 50 (variations of tripod grasp), where the difference between the baseline-1 and the WD, MW augmentations is bigger. Similarly, in putEMG the most gain in classification is observed in labels 4–7 that correspond to a subject performing a pinch grasp using the thumb and any of the other fingers. This indicates that the augmentation methods investigated in this study improve the performance on difficult gestures.

Considering that the *WeiNet* model has far more weights than the AtzoriNet*, we did not perform a grid search for the optimum augmentation hyper-parameters. Rather, based on the results from AtzoriNet*, the best augmentation methods, namely the WD and MW, are applied. As reported in [19], the performance of the model on the Ninapro dataset when maximum overlap is used, i.e., SW(01), is 0.85. Due to differences in the development tools, the baseline accuracy we achieved is 0.8480. Table A14 shows that, with the MW augmentation, the baseline performance is improved by 1% when the augmentation ratio is set to R=15. A repeated measures ANOVA test showed that this change in accuracy is significant (p<0.001). Additionally, the WD method performed worse than the baseline, which consequently had a negative effect on the accuracy of WD+MW.

## 6. Conclusions

This work explored the application of data augmentation techniques on sEMG signals for the problem of gesture recognition. Five approaches, i.e., SS1/2, WD, MW, GN, and SW, were evaluated with different amounts of augmentation *R* across two datasets. The evaluation was based on the AtzoriNet*, a modified version of a lightweight CNN for gesture recognition, while the findings were also applied to the state-of-the-art model in the domain (*Weinet* [19]). The results showed that the recognition accuracy never decreases significantly except in one case (i.e., in the SS1 method on Ninapro dataset), while it can be improved if the training set is inflated with generated signals that provide adequate data diversity for training DL models. Simply by generating the input images using overlapping windows (i.e., SW(01) method), the gain in accuracy is significantly larger than the non-overlapping case (i.e., SW(15) method), which is further significantly improved with the MW or WD methods on the Ninapro dataset. Selecting the appropriate amount of augmentation is important since the required data size depends on the dataset and the model architecture. Finally, the classification accuracy of the state-of-the-art model was significantly improved by 1% using the investigated augmentation methods.

## Appendix A

**Table A1:** Architecture details of the two CNN models.

Model	*AtzoriNet** [22]	*WeiNet* [19]
Details		s = {
		Conv2D(64, (3,3))
	Conv2D(32, (1,$C))	Conv2D(64, (3,3))
	Conv2D(32, (3,3))	LC(64, (1,1))
	MaxPool(3,3)	LC(64, (1,1))
	Conv2D(64, (5,5))	}× $C
	MaxPool(3,3)	Concat(s)
	Conv2D(64, (5,1))	FC(512)
	Conv2D($G, (1,1))	FC(512)
	Softmax	FC(128)
		FC($G)
		Softmax
Parameters	84K	8M

$C: number of sEMG channels, $G: number of gestures, $\mathrm{Conv}2D(k,\left(f_{1},f_{2}\right))$: 2D Convolutional layer of *k* filters with size $\left(f_{1},f_{2}\right)$, $\mathrm{MaxPool}(f_{1},f_{2})$: max pooling layer of size $\left(f_{1},f_{2}\right)$, $\mathrm{LC}(k,\left(f_{1},f_{2}\right))$: locally connected layer of *k* filters of size $\left(f_{1},f_{2}\right)$, Concat(s): layer that concatenates a list of feature maps *s* along the last axis, FC(k): fully connected layer of *k* units, *Softmax*: softmax distribution layer.

**Table A2:** Hyper-parameters used for the optimization of the CNN models.

Model	*AtzoriNet** [22]	*WeiNet* [19]
Optimizer	Adam	SGD with momentum
Learning rate	0.001	0.1
Learning rate schedule	constant	divide by 10 after epochs 1624
Weight decay	0.0005	0.0001
Epochs	100 (early stopping)	28 (preceded by pretraining)
Batch size	512	1024

**Table A3:** Datasets details and partition into train, validation and test sets based on gesture repetitions.

Dataset	Sampling (Hz)	sEMG Channels	Subjects	Gestures	Trials	Train	Validation	Test
Ninapro-DB1 [23]	100	10	27	52+1	10	1, 3, 4, 6, 8, 9, 10	9, 10	2, 5, 7
putEMG [25]	5124	3×8	44	7+1	8	1, 3, 4, 6, 7, 8	8	2, 5

**Table A4:** Augmentation hyper-parameter selection and search space for each method. The selected values are shown for each dataset.

Method	Hyper-Parameter	Search Space	Selected Value (DB1/putEMG)
WD	ψ	{coif2, db7, sym4}	sym4/db7
	*l*	{4, 5}	5
	*b*	{0, 2–5}	3/0
MW	σ	{0.1, 0.2, 0.3}	0.1
GN	SNR	{25, 30, 35}	30/35

**Table A5:** Augmentation hyper-parameter selection results. For each method and dataset, higher accuracy is shown in bold.

Method	Hyper-Parameter Value	Accuracy Ninapro-DB1	Accuracy putEMG
GN	SNR=25	0.5862	0.8150
GN	SNR=30	**0.6007**	0.8509
GN	SNR=35	0.5994	**0.8551**
MW	σ=0.1	**0.6342**	**0.8529**
MW	σ=0.2	0.6123	0.8210
MW	σ=0.3	0.5904	0.7486
WD	ψ=coif2,l=4,b=0	0.6145	0.8377
WD	ψ=coif2,l=4,b=2	0.6068	0.8576
WD	ψ=coif2,l=4,b=3	0.6074	0.8456
WD	ψ=coif2,l=4,b=4	0.6064	0.8629
WD	ψ=coif2,l=4,b=5	0.5890	0.8763
WD	ψ=coif2,l=5,b=0	0.6112	0.8611
WD	ψ=coif2,l=5,b=2	0.6152	0.8664
WD	ψ=coif2,l=5,b=3	0.6129	0.8752
WD	ψ=coif2,l=5,b=4	0.5985	0.8473
WD	ψ=coif2,l=5,b=5	0.5764	0.8383
WD	ψ=db7,l=4,b=0	0.6127	0.8454
WD	ψ=db7,l=4,b=2	0.6120	0.8592
WD	ψ=db7,l=4,b=3	0.6068	0.8617
WD	ψ=db7,l=4,b=4	0.5907	0.8669
WD	ψ=db7,l=4,b=5	0.5751	0.8391
WD	ψ=db7,l=5,b=0	0.6168	**0.8770**
WD	ψ=db7,l=5,b=2	0.6142	0.8429
WD	ψ=db7,l=5,b=3	0.6036	0.8714
WD	ψ=db7,l=5,b=4	0.5858	0.8635
WD	ψ=db7,l=5,b=5	0.5634	0.8213
WD	ψ=sym4,l=4,b=0	0.6146	0.8567
WD	ψ=sym4,l=4,b=2	0.6149	0.8640
WD	ψ=sym4,l=4,b=3	0.6129	0.8446
WD	ψ=sym4,l=4,b=4	0.6072	0.8757
WD	ψ=sym4,l=4,b=5	0.6008	0.8632
WD	ψ=sym4,l=5,b=0	0.6162	0.8579
WD	ψ=sym4,l=5,b=2	0.6122	0.8759
WD	ψ=sym4,l=5,b=3	**0.6240**	0.8372
WD	ψ=sym4,l=5,b=4	0.6004	0.8521
WD	ψ=sym4,l=5,b=5	0.5846	0.8590

**Table A6:** Average accuracy of *AtzoriNet** for each method at different augmentation ratios, *R*, for the Ninapro-DB1 dataset. The value in bold corresponds to the method with highest accuracy.

R	SS1	SS2	WD	MW	GN	WD+MW	AA	AO	AR
1	0.6937	0.7262	0.7308	0.7337	0.7306	0.7354	0.7284	0.7312	0.7322
4	0.6332	0.7166	0.7405	0.7436	0.7251	0.7389	0.7391	0.7377	0.7399
9	0.5786	0.7152	0.7345	**0.7443**	0.7331	0.7399	0.7256	0.7405	0.7383
SW(15) (baseline-1): 0.6305, SW(01) (baseline-2): 0.7273									

**Table A7:** Average accuracy of *AtzoriNet** for each method at different augmentation ratios, *R*, for the putEMG dataset. The value in bold corresponds to the method with highest accuracy.

R	SS1	SS2	WD	MW	GN	WD+MW	AA	AO	AR
1	0.9442	0.9498	0.9474	0.9477	0.9496	0.9506	0.9423	0.9453	0.9507
4	0.9500	0.9622	0.9660	0.9580	0.9569	0.9571	0.9489	0.9621	0.9616
9	0.9471	0.9584	0.9586	0.9605	0.9616	0.9612	0.9498	0.9696	0.9660
15	0.9406	0.9613	0.9602	0.9650	0.9654	0.9606	0.9482	**0.9697**	0.9672

SW(15) (baseline-1): 0.8071, SW(01) (baseline-2): 0.9388.

**Table A8:** Average and standard deviation across subjects of the cluster metrics Silhouette Coefficient (SC), Davies–Bouldin (DB) and classification accuracy (row of R=9 in Table A6) for the Ninapro-DB1 dataset.

Method	SC	DB	Accuracy
SW(15) (baseline-1)	0.2089 ± 0.0498	1.67 ± 0.21	0.6305 ± 0.0608
SW(01) (baseline-2)	0.2783 ± 0.0461	1.45 ± 0.17	0.7273 ± 0.0581
SS1(01)	0.1535 ± 0.0395	1.90 ± 0.22	0.5786 ± 0.0594
SS2(01)	0.2980 ± 0.0486	1.36 ± 0.16	0.7152 ± 0.0564
WD(01)	0.2627 ± 0.0468	1.47 ± 0.18	0.7345 ± 0.0591
MW(01)	0.2768 ± 0.0459	1.42 ± 0.16	0.7443 ± 0.0558
GN(01)	0.2814 ± 0.0492	1.42 ± 0.18	0.7331 ± 0.0618
WD+MW(01)	0.2686 ± 0.0428	1.44 ± 0.15	0.7399 ± 0.0594
AA(01)	0.2600 ± 0.0430	1.47 ± 0.15	0.7256 ± 0.0587
AO(01)	0.2838 ± 0.0465	1.40 ± 0.17	0.7405 ± 0.0588
AR(01)	0.2842 ± 0.0468	1.41 ± 0.17	0.7383 ± 0.0591

**Table A9:** Average and standard deviation across subjects of the cluster metrics Silhouette Coefficient (SC), Davies–Bouldin (DB) and classification accuracy (row of R=15 in Table A7) for the putEMG dataset.

Method	SC	DB	Accuracy
SW(15) (baseline-1)	0.3349 ± 0.0732	1.58 ± 0.55	0.8071 ± 0.0933
SW(01) (baseline-2)	0.4998 ± 0.0648	0.94 ± 0.22	0.9388 ± 0.0602
SS1(01)	0.4287 ± 0.0903	1.11 ± 0.40	0.9406 ± 0.0480
SS2(01)	0.5295 ± 0.0685	0.78 ± 0.16	0.9613 ± 0.0375
WD(01)	0.5292 ± 0.0654	0.79 ± 0.16	0.9602 ± 0.0448
MW(01)	0.4773 ± 0.0743	0.91 ± 0.22	0.9650 ± 0.0356
GN(01)	0.5289 ± 0.0734	0.80 ± 0.19	0.9654 ± 0.0433
WD+MW(01)	0.4827 ± 0.0783	0.90 ± 0.21	0.9606 ± 0.0464
AA(01)	0.4728 ± 0.0755	0.94 ± 0.23	0.9482 ± 0.0528
AO(01)	0.5261 ± 0.0683	0.80 ± 0.18	0.9697 ± 0.0299
AR(01)	0.5394 ± 0.0603	0.77 ± 0.15	0.9672 ± 0.0384

**Table A10:** Pairwise comparisons with Bonferonni correction for the Ninapro-DB1 dataset. The table shows the *p*-value of the comparison between the classification accuracy of the methods on the corresponding row and column. Values above the diagonal show the comparisons for R=9 and below the diagonal for R=1 (last and first row of Table A6, respectively). Values in bold correspond to the comparisons of the method with the highest classification accuracy in Table A6 (i.e., MW). An ‘*’ denotes a significant difference (α=0.05), while a ‘**’ denotes a *p*-value p<0.001.

	SW(15)	SW(01)	SS1(01)	SS2(01)	WD(01)	MW(01)	GN(01)	WD+MW(01)	AA(01)	AO(01)	AR(01)
SW(15)	—	**	**	**	**	******	**	**	**	**	**
SW(01)	**	—	**	0.009 *	0.590	******	1.0	0.019 *	1.0	0.004 *	0.004 *
SS1(01)	**	**	—	**	**	******	**	**	**	**	**
SS2(01)	**	1.0	**	—	**	******	**	**	0.029*	**	**
WD(01)	**	1.0	**	1.0	—	**0.007 ***	1.0	1.0	0.209	1.0	1.0
MW(01)	**	1.0	**	0.270	1.0	—	**0.064**	**1.0**	******	**1.0**	**0.739**
GN(01)	**	1.0	**	1.0	1.0	1.0	—	1.0	0.554	0.123	1.0
WD+MW(01)	**	0.076	**	0.002 *	0.866	1.0	1.0	—	**	1.0	1.0
AA(01)	**	1.0	**	1.0	1.0	0.617	1.0	0.086	—	**	0.001 *
AO(01)	**	1.0	**	1.0	1.0	1.0	1.0	1.0	1.0	—	1.0
AR(01)	**	1.0	**	0.560	1.0	1.0	1.0	1.0	1.0	1.0	—

**Table A11:** Pairwise comparisons with Bonferonni correction for the putEMG dataset. The table shows the *p*-value of the comparison between the classification accuracy of the methods on the corresponding row and column. Values above the diagonal show the comparisons for R=15 and below the diagonal for R=1 (last and first row of Table A7, respectively). Values in bold correspond to the comparisons of the method with the highest classification accuracy in Table A7 (i.e., AO). An ‘*’ denotes a significant difference (α=0.05), while a ‘**’ denotes a *p*-value p<0.001.

	SW(15)	SW(01)	SS1(01)	SS2(01)	WD(01)	MW(01)	GN(01)	WD+MW(01)	AA(01)	AO(01)	AR(01)
SW(15)	—	**	**	**	**	**	**	**	**	******	**
SW(01)	**	—	1.0	0.269	0.016*	0.003 *	**	0.022 *	1.0	**0.003 ***	0.006 *
SS1(01)	**	1.0	—	0.102	0.250	0.003 *	0.040 *	0.604	1.0	******	0.002 *
SS2(01)	**	1.0	1.0	—	1.0	1.0	1.0	1.0	1.0	**0.572**	1.0
WD(01)	**	1.0	1.0	1.0	—	1.0	1.0	1.0	1.0	**0.840**	1.0
MW(01)	**	1.0	1.0	1.0	1.0	—	1.0	1.0	0.415	**1.0**	1.0
GN(01)	**	0.050	1.0	1.0	1.0	1.0	—	1.0	0.564	**1.0**	1.0
WD+MW(01)	**	0.756	1.0	1.0	1.0	1.0	1.0	—	1.0	**1.0**	1.0
AA(01)	**	1.0	1.0	1.0	1.0	1.0	1.0	1.0	—	**0.002 ***	0.001 *
AO(01)	**	1.0	1.0	1.0	1.0	1.0	1.0	1.0	1.0	—	**1.0**
AR(01)	**	1.0	1.0	1.0	1.0	1.0	1.0	1.0	1.0	1.0	—

**Table A12:** Pairwise comparisons with Bonferonni correction for the Ninapro-DB1 dataset. The table shows the *p*-value of the comparison between the clustering metrics of the methods on the corresponding row and column. Values above the diagonal show the comparisons for the SC metrics and below the diagonal for the DB metrics (second and third columns of Table A8, respectively). An ‘*’ denotes a significant difference (α=0.05), while a ‘**’ denotes a *p*-value p<0.001.

	SW(15)	SW(01)	SS1(01)	SS2(01)	WD(01)	MW(01)	GN(01)	WD+MW(01)	AA(01)	AO(01)	AR(01)
SW(15)	—	**	**	**	**	**	**	**	**	**	**
SW(01)	**	—	**	**	**	1.0	1.0	0.186	**	1.0	0.449
SS1(01)	**	**	—	**	**	**	**	**	**	**	**
SS2(01)	**	**	**	—	**	**	**	**	**	**	**
WD(01)	**	1.0	**	**	—	0.060	**	1.0	1.0	**	**
MW(01)	**	1.0	**	0.016 *	0.220	—	1.0	0.005 *	**	1.0	1.0
GN(01)	**	0.379	**	0.007 *	0.001 *	1.0	—	0.011 *	**	1.0	1.0
WD+MW(01)	**	1.0	**	**	1.0	0.703	1.0	—	0.087	**	**
AA(01)	**	1.0	**	**	1.0	0.004 *	0.009 *	0.324	—	**	**
AO(01)	**	0.005 *	**	0.029 *	**	1.0	1.0	0.014 *	**	—	1.0
AR(01)	**	0.001 *	**	0.002 *	**	1.0	1.0	0.037 *	**	1.0	—

**Table A13:** Pairwise comparisons with Bonferonni correction for the putEMG dataset. The table shows the *p*-value of the comparison between the clustering metrics of the methods on the corresponding row and column. Values above the diagonal show the comparisons for the SC metrics and below the diagonal for the DB metrics (second and third columns of Table A9, respectively). An ‘*’ denotes a significant difference (α=0.05), while a ‘**’ denotes a *p*-value p<0.001.

	SW(15)	SW(01)	SS1(01)	SS2(01)	WD(01)	MW(01)	GN(01)	WD+MW(01)	AA(01)	AO(01)	AR(01)
SW(15)	—	**	**	**	**	**	**	**	**	**	**
SW(01)	**	—	**	**	0.001 *	0.345	**	1.0	0.086	0.030 *	**
SS1(01)	**	0.082	—	**	**	0.002 *	**	**	0.006 *	**	**
SS2(01)	**	**	**	—	1.0	**	1.0	**	**	1.0	1.0
WD(01)	**	**	**	1.0	—	**	1.0	**	**	1.0	1.0
MW(01)	**	1.0	0.008 *	**	0.009 *	—	**	1.0	1.0	**	**
GN(01)	**	**	**	1.0	1.0	0.029 *	—	**	**	1.0	1.0
WD+MW(01)	**	1.0	0.008 *	**	0.003 *	1.0	0.036 *	—	1.0	**	**
AA(01)	**	1.0	0.022 *	**	**	1.0	0.003 *	1.0	—	**	**
AO(01)	**	**	**	1.0	1.0	**	1.0	0.001 *	**	—	0.329
AR(01)	**	**	**	1.0	1.0	**	1.0	**	**	1.0	—

**Table A14:** Average accuracy of *WeiNet* for each method at different augmentation ratios, *R*, for the Ninapro-DB1 dataset. The value in bold corresponds to the highest accuracy.

R	WD	MW	WD + MW
1	0.8439	0.8514	0.8498
4	0.8413	0.8556	0.8493
9	0.8421	0.8573	0.8541
15	0.8239	**0.8581**	0.8511
SW(01) (baseline-2): 0.8480.			
